# Quantitative genetics of taura syndrome resistance in pacific white shrimp (*penaeus vannamei*): a cure model approach

**DOI:** 10.1186/1297-9686-43-14

**Published:** 2011-03-21

**Authors:** Jørgen Ødegård, Thomas Gitterle, Per Madsen, Theo HE Meuwissen, M Hossein Yazdi, Bjarne Gjerde, Carlos Pulgarin, Morten Rye

**Affiliations:** 1 Nofima Marin, NO-1432 Ås, Norway; 2 Norwegian University of Life Sciences, NO-1432 Ås, Norway; 3 Akvaforsk Genetics Center AS, NO-6600 Sunndalsøra, Norway; 4 CENIACUA, Bogotá, Colombia; 5 Aarhus University, DK-8830 Tjele, Denmark

## Abstract

**Background:**

In aquaculture breeding, resistance against infectious diseases is commonly assessed as time until death under exposure to a pathogen. For some diseases, a fraction of the individuals may appear as "cured" (non-susceptible), and the resulting survival time may thus be a result of two confounded underlying traits, i.e., endurance (individual hazard) and susceptibility (whether at risk or not), which may be accounted for by fitting a cure survival model. We applied a cure model to survival data of Pacific white shrimp (*Penaeus vannamei*) challenged with the Taura syndrome virus, which is one of the major pathogens of Panaeid shrimp species.

**Methods:**

In total, 15,261 individuals of 513 full-sib families from three generations were challenge-tested in 21 separate tests (tanks). All challenge-tests were run until mortality naturally ceased. Time-until-event data were analyzed with a mixed cure survival model using Gibbs sampling, treating susceptibility and endurance as separate genetic traits.

**Results:**

Overall mortality at the end of test was 28%, while 38% of the population was considered susceptible to the disease. The estimated underlying heritability was high for susceptibility (0.41 ± 0.07), but low for endurance (0.07 ± 0.03). Furthermore, endurance and susceptibility were distinct genetic traits (r_g _= 0.22 ± 0.25). Estimated breeding values for endurance and susceptibility were only moderately correlated (0.50), while estimated breeding values from classical models for analysis of challenge-test survival (ignoring the cured fraction) were closely correlated with estimated breeding values for susceptibility, but less correlated with estimated breeding values for endurance.

**Conclusions:**

For Taura syndrome resistance, endurance and susceptibility are apparently distinct genetic traits. However, genetic evaluation of susceptibility based on the cure model showed clear associations with standard genetic evaluations that ignore the cure fraction for these data. Using the current testing design, genetic variation in observed survival time and absolute survival at the end of test were most likely dominated by genetic variation in susceptibility. If the aim is to reduce susceptibility, earlier termination of the challenge-test or back-truncation of the follow-up period should be avoided, as this may shift focus of selection towards endurance rather than susceptibility.

## Background

Genetic evaluation of resistance against infectious diseases in aquaculture species is typically based on data from challenge-tests, where individuals are exposed to the relevant pathogen under controlled environmental conditions. Traditionally, such evaluations have been based on cross-sectional models, i.e., models considering survival as an all-or-non trait (survived/dead at a specific point in time). More recent studies in aquaculture species have suggested using more advanced longitudinal survival models [1-3], such as proportional hazards frailty models [4] or survival score models [5]. These models take into account not only whether the individual survives a given time period, but also time until death. A typical assumption in survival analysis is that all individuals are at risk, i.e., censored lifespans are simply the result of a limited follow-up period. However, this assumption is violated if a fraction of the individuals are non-susceptible (e.g., not infected or tolerant), which is not unlikely when testing for resistance against specific pathogens [e.g., [6,7]]. Given that a fraction of non-susceptible individuals exists, mortality is expected to level out when the majority of the susceptible individuals have died, rather than approaching 100%.

Genetic evaluations of binary traits are expected to be most accurate at intermediate frequencies [8]. To achieve this, challenge-tests in aquaculture breeding programs have often been terminated at intermediate but still increasing mortalities, or evaluation datasets have been back-truncated at such frequencies. However, this would only be an advantage when analyzing survival data with cross-sectional models that treat survival as a binary trait. For classical longitudinal survival models, high mortality (and thus limited censoring) would be an advantage in genetic analysis [9]. Furthermore, the practice of early termination or back-truncation is based on the assumption that survival time and long-term survival under exposure to the pathogen are equivalent genetic traits. Given the presence of non-susceptible individuals, this may not be the case. For example, in wild Atlantic salmon, some Baltic populations are to a large extent tolerant to the ectoparasite *Gyrodactylus salaris*, while East Atlantic stocks are highly susceptible [10,11], leading to mass mortalities in infected rivers [12]. Hence, comparing these populations on survival time would be inappropriate. Furthermore, even within a highly susceptible Norwegian river population, a small fraction of long-term survivors was identified. In the latter population, susceptibility (long-term survival) and endurance (time until death of non-survivors) appeared to have a low genetic correlation, indicating that these two aspects of parasite resistance are genetically distinct traits [12].

Given that a non-susceptible fraction exists and that endurance and susceptibility are distinct genetic traits, selection programs for improved disease resistance would (if given the opportunity) most likely favor improvement of non-susceptibility over endurance, as the latter may postpone mortality rather than avoid it in the long run. Existence of non-susceptible individuals may also reduce pathogenic pressure in the population, while highly endurant (but infected) individuals may produce large numbers of infectious disease agents during their long period of infection. Still, in real disease testing schemes, follow-up periods are often limited due to practical considerations, and survivors are thus expected to be a mixture of non-susceptible and susceptible individuals with censored lifespans. A mixture cure model [13] is a survival model that attempts to distinguish susceptible and non-susceptible ("cured") survivors, which may be of great advantage in the analysis of time-to-event data that contain a cure fraction.

Taura syndrome (TS) is an economically important viral disease affecting Panaeid shrimp and has been responsible for mass mortality in Pacific white shrimp (*Penaeus vannamei*). The Taura syndrome virus (TSV) was first discovered in South America, but has later spread to North America, Hawaii and Asia [14-16]. A substantial underlying heritability (0.30 ± 0.13) has been estimated for TS resistance, and selective breeding is successfully implemented; i.e., survival after exposure to TSV increased by 18.4% after only one generation of selection for TS resistance [17]. Furthermore, in a Colombian mass selection program for TS resistance, overall survival in TSV infected areas is now back to the levels prior to the first outbreak of TS [18].

The aim of the study was to apply a cure model to survival data from challenge testing of Pacific white shrimp with TSV and to compare this model with classical models of analysis of such data.

## Methods

### Data

**Figure 1:**
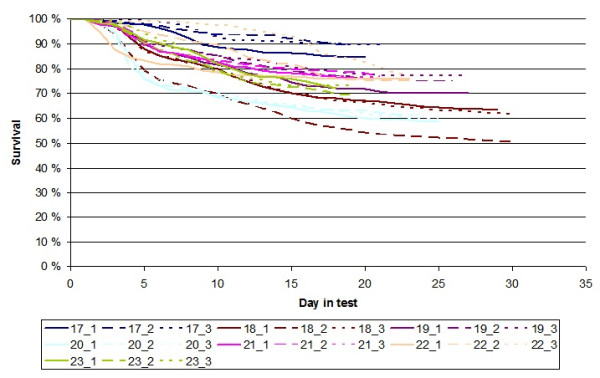
**Kaplan-Meier survival curves for the different TSV challenge tests**. The different challenge tests are numbered as "batch_tank" (batches 17 to 23).

The study was based on recorded survival times of 15,261 Pacific white shrimp from Colombia. The shrimp originated from 513 full-sib families (266 sires and 484 dams). The parents were selected for TS resistance and growth through a combined individual and family-based selection program [18]. The dataset contained individuals from seven different batches, including three consecutive generations. Parents were used across several batches, resulting in good genetic ties between the different groups in the dataset. All families were kept separate in different tanks until they were individually tagged when the population reached the average size of one gram (normally eight weeks after hatching). Animals from the same full-sib family were randomly selected and tagged with a common color code by injecting differently colored fluorescent elastomers into the 6th abdominal segment of each animal. Each batch was tested separately in three different test-tanks. Shrimp from the first batch were orally infected with TSV-infected minced muscle tissue for seven consecutive days at a feeding intensity of 10% of the tank biomass per day. Due to low mortality, the animals of the second batch onward were infected through intramuscular injections of 20 μL of a purified inoculum of the pathogen. For each test, mortalities were recorded on an hourly basis until no more dead animals were recorded for 24 hours. The length of the recording periods in the different test lasted from 18 to 30 days (Figure 1).

### Statistical analysis

Survival times in hours were transformed to test-day (24 h) binary survival scores. Hence, the number of records per individual equals the number of days (measured from the time of the first observed mortality in the test) until death or censoring. For each period, an individual was scored as dead (= 1) if it was recorded as dead during that period and as alive (= 0) otherwise, e.g., an animal dying at day 4 had survival scores of [0 0 0 1]. The model and Bayesian setup is described in more detail by Ødegård *et al*.[19]. Here, the probability of an individual *i *being censored (*c_i _*= 1) at the end of the follow-up period (survival time *y_i _*= *t*) is:(1)

where *Z_i _*is the susceptibility status (susceptible = 1; non-susceptible = 0), *S_ij _*is the endurance score for period *j*, i.e. whether individual *i *survives (0) or dies (1) in time period *j*, given that it is susceptible.

Given *Z *and *S*, the cure model is reduced to a bivariate threshold model, where the first trait (susceptibility status, *Z*) is whether the animal is susceptible to the disease, and the second trait consists of endurance scores (*S*), which are only observable for putatively susceptible animals (*Z_i _*= 1). The corresponding underlying liabilities of the two traits were analyzed with the following model (CURE):(2)

where **λ**_**1 **_and **λ**_**2 **_are vectors of liabilities associated with endurance scores and susceptibility statuses, respectively,  is a vector of "fixed" effects (batch-tank and overall mean for endurance and susceptibility, respectively),  is a vector of random (batch-tank) test-day effects on **λ**_**1**_, with variance ,  is a vector of random batch-tank effects on **λ**_**2**_, with variance ,  is a vector of random additive genetic effects of all individuals,  is a vector of random common environmental family effects (i.e., potential effects of separate rearing of families prior to tagging, maternal effects and dominance genetic effects),  is a vector of random residuals associated with both traits, **G **is the genetic co-variance matrix, **F **is the co-variance matrix of common environmental family effects, **A **is the additive genetic relationship matrix and **I **denotes an identity matrix of appropriate size. As endurance can only be observed in putatively susceptible individuals, the residual covariance between the two underlying traits is not identifiable and was restricted to be zero [19], as indicated above.

In the CURE model, the susceptibility status (*Z_i_*) of a survivor *i*, surviving *t *days in a given test was sampled from a Bernoulli distribution with a conditional probability for susceptibility [19] calculated as:(3)

where **θ **is a vector of all location parameters, the **w' **vectors are appropriate row incidence vectors associated with the location parameters of the endurance and susceptibility liabilities of the individual. The standard normal cumulative density function  is thus the prior probability of being susceptible (*Z_i _*= 1) for individual *i *(given the model parameters) and  is the probability (given the model parameters) for individual *i *to survive until day *t *(end of test), given that the individual is susceptible. Based on observed survival time and the sampled putative susceptibility status, we defined a set of putative "endurance scores", which were defined based on the recorded survival time and censoring status (as described above) for the putatively susceptible individuals, and defined as missing for the putatively non-susceptible ones (as endurance does not influence survival time in non-susceptible animals). Given the endurance scores and the susceptibility statuses, all parameters of the CURE model were sampled as in a standard bivariate threshold model using Gibbs sampling.

For comparison purposes the survival data were also fitted using a "naïve" (assuming that all individuals are susceptible) survival score threshold model (NAÏVE) and a simple cross-sectional threshold model for observed survival until the end of test (SIMPLE).

The NAÏVE model was:(4)

where **λ_1 _**is a vector of liabilities associated with the survival scores, and the other parameters are as described above.

The SIMPLE model was:(5)

where **λ_2 _**is a vector of liabilities associated with observed survival to the end of test, **μ**_**2 **_is a vector of fixed batch-tank effects and the other parameters are as described above. To avoid bias problems typical of animal threshold models [20], genetic (co)variance components were estimated with an algorithm that was based on parental breeding values only [21], while all other dispersion and location parameters were estimated as in a standard animal threshold model.

All genetic analyses were performed using a modified Gibbs sampling module in the DMU software package [22]. Convergence was checked through visual inspection of trace plots and Raftery and Lewis diagnostics [23]. The NAÏVE and SIMPLE models were run in single chains for 110,000 rounds, discarding the initial 10,000 rounds as burn-in, and storing parameters of every 10^th ^sampling round. The CURE model had considerably slower mixing compared with the SIMPLE and NAÏVE models and two separate longer chains were thus chosen for this model (2×340,000 rounds, discarding the first 40,000 as burn-in). Two separate chains rather than one long chain were chosen to reduce the computing time (which varied between 82 h to 123 h), and results were averaged across the two chains. Due to limitations in storing capacity, samples from every 100 rounds were kept for the latter model.

## Results

Descriptive statistics of the data set are given in Table 1, and Kaplan-Meier survival curves for the different tanks and batches are given in Figure 1. Across challenge-tests, the average mortality was 28% but varied substantially between tests. Environment and management (water temperature and tank densities) were standardized across tanks and batches to achieve as high as possible mortality during the testing period, and no clear phenotypic trends over generations and batches were therefore evident. Even though the tests lasted until mortality naturally stopped, survival was above 50% in all challenge tests. Furthermore, most survival curves showed a clear tendency towards flattening out at moderate or high frequencies, which is consistent with a substantial fraction of non-susceptible individuals in the population.

**Table 1:** Descriptive statistics of the data set

Item	
Shrimp with data	15,261
Full-sib families	513
Sires	266
Dams	484
Generations with data	3
Batches with data	7
Challenge-test tanks per batch	3
Average mortality (across tests)	28%
Median time until death^1 ^(across tests)	157 h (56 h)

^1^Excluding individuals with censored lifespans. Between-test standard deviation is presented in parenthesis.

Results of the current analyses are presented in Table 2. Based on the SIMPLE model, the underlying heritability of end-survival was substantial (h^2 ^= 0.39 ± 0.06). The fraction of underlying variance explained by common environmental effects was small (c^2 ^= 0.05 ± 0.02) but significant (based on a likelihood ratio test, using a linear model). Likewise, the NAÏVE model also indicated moderate heritable variation for endurance, with an estimated underlying heritability of 0.16 ± 0.03 for test-day endurance scores, while common environmental family effects explained only a minor part of the total underlying variance for endurance scores (c^2 ^= 0.02 ± 0.01). The estimated test-day (environmental) variance was rather small and explained only 9% of the underlying variation in endurance liability (posterior mean).

**Table 2:** Posterior means of parameters for the CURE, SIMPLE and NAÏVE threshold models (± posterior standard deviations)

**Parameters**^**1**^	Trait	CURE	SIMPLE	NAÏVE
Fraction susceptible (%)		38 ± 1	-	100
	Endurance/survival scores	0.14 ± 0.01	-	0.13 ± 0.01
	Endurance/survival scores	0.09 ± 0.04	-	0.21 ± 0.05
	Susceptibility/mortality	0.82 ± 0.23	0.72 ± 0.16	-
*r_g_*	Endurance - susceptibility	0.22 ± 0.25	-	-
	Endurance/survival scores	0.07 ± 0.02	-	0.03 ± 0.01
	Susceptibility/mortality	0.13 ± 0.04	0.08 ± 0.03	
*r_f _*	Endurance - susceptibility	-0.06 ± 0.05	-	-
	Susceptibility	0.16 ± 0.10	-	-
*h*^2 ^	Endurance/survival scores^2^	0.07 ± 0.03	-	0.16 ± 0.03
	Susceptibility/mortality^3^	0.41 ± 0.07	0.39 ± 0.06	-
*c^2 ^*	Endurance/survival scores^4^	0.05 ± 0.01	-	0.02 ± 0.01
	Susceptibility/mortality^5^	0.07 ± 0.02	0.05 ± 0.02	-

^1 ^ = variance of tank-test-day effects,  = genetic variance, *r*_*g *_= genetic correlation,  = variance of common environmental family effects, *r_f _*= correlation of common environmental family effects,  = variance of batch-tank effects,

^2^, ^3^

^4 ^, ^5 ^

For the CURE model, the posterior mean of the percentage of putative susceptible shrimp was 38% (± 1%), while 28% of the shrimp actually died (Table 1). Hence, across tests, 86% ((1-0.38)/(1-0.28) = 0.86) of the survivors were considered as non-susceptible. For the CURE model, the estimated underlying heritability (h^2 ^= 0.07 ± 0.03) for endurance was smaller than for the NAÏVE model, while the estimated underlying heritability of susceptibility was similar (h^2 ^= 0.41 ± 0.07) to the estimated heritability for end-survival for the SIMPLE model. The genetic correlation between endurance and susceptibility within the CURE model tended to be positive but not significantly different from zero (r_g _= 0.22 ± 0.25). Furthermore, the sampled genetic correlation between endurance and susceptibility was lower than 0.8 in 99% of the sampling rounds of the Gibbs chain, indicating that endurance and susceptibility should be considered as distinct genetic traits. As for the other models, common environmental effects explained a relatively small part of the underlying liability variance for both endurance and susceptibility for the CURE model (c^2 ^= 5% and c^2 ^= 7%, respectively). The correlation between common environmental effects on the two traits was low (r_f _= -0.06 ± 0.05). Finally, the random tank-test-day effects for endurance and the random batch-tank effects for susceptibility explained a relatively small fraction of the underlying liability variances (posterior means of 11% and 7%, respectively).

Table 3 shows the Pearson and Spearman correlation coefficients between predicted breeding values (EBV) from the three models. Correlations between EBV of the SIMPLE and NAÏVE models were close to unity (0.99), and both models showed very high correlations (0.98-0.99) with the EBV of susceptibility in the CURE model; while the correlations with the endurance EBV were substantially lower (0.57-0.63). Similarly, the EBV for endurance and susceptibility from the CURE model were only moderately correlated to each other (0.50-0.51).

**Table 3:** Pearson (above diagonal) and Spearman (below diagonal) correlation coefficients between posterior means of breeding values for the different models

Model		CURE		SIMPLE	NAÏVE
	Trait	Endurance	Susceptibility	End-survival	Survival
CURE	Endurance		0.51	0.57	0.63
	Susceptibility	0.50		0.99	0.98
SIMPLE	End-survival	0.57	0.99		0.99
NAÏVE	Survival	0.61	0.98	0.99	

## Discussion

The estimated underlying heritability of end-survival using the SIMPLE model was substantial (0.39 ± 0.06). This is in line with previously reported estimates of heritability for survival to TS (0.30 ± 0.13) from a different population of Pacific white shrimp [17]. The NAÏVE model also indicated considerable heritable variation for survival scores, but lower than for end-survival. This was expected, as the model splits the lifespan in several shorter periods. The estimated underlying heritability of susceptibility from the CURE model was similar (0.41 ± 0.07) to the estimated heritability of end-survival from the SIMPLE model, which may be due to the fact that challenge-tests were continued until mortality naturally ceased. Hence, few susceptible individuals were likely to survive, which is supported by the high fraction of putatively "cured" animals among the survivors in the CURE model (86%). The estimated heritability for endurance from the CURE model was about half the corresponding heritability of the NAÏVE model, i.e., in a standard survival model, the more highly heritable susceptibility status is likely to dominate survival time and thereby increase the estimated genetic variance.

Based on the results from the CURE model, endurance and susceptibility appear to be distinct genetic traits with respect to TS resistance. If the aim is to improve long-term survival to TS in the population, selection for increased time until death or survival to the end of test is therefore likely to be suboptimal, and more so if testing is based on data from challenge-tests with short follow-up periods or survival data that are back-truncated to a point in time where mortality is still increasing. In a simulation study, it was concluded that if selection aims at improving susceptibility the error of applying a classical "non-cure" survival model was non-neglible, especially if endurance and susceptibility were lowly genetically correlated and when genetic variance of endurance is substantial [19]. However, by using the current testing strategy where the testing period continues until mortality naturally ceases, there were small practical differences between selection for increased survival using classical models (SIMPLE and NAÏVE) and the more advanced CURE model. Hence, correlations between EBV of the SIMPLE and NAÏVE models were close to unity, and both models showed good agreement with the EBV for susceptibility from the CURE model. Still, the EBV for endurance from the CURE model were only moderately correlated to EBV for susceptibility from that same model (and to EBV for the SIMPLE and NAÏVE models). These results indicate that genetic variation in recorded end-survival and time until death of the current data set are dominated by genetic variation in susceptibility. Furthermore, stopping the test at an earlier stage would shift the focus of selection towards endurance (especially for classical models).

In a theoretical study, Ødegård *et al*. [19] have shown that a truly positive genetic correlation between endurance and susceptibility in a cure model may be underestimated as result of large uncertainty (giving more room for downward than upward errors). This could in part explain the low genetic correlation obtained for endurance and susceptibility in this study. Still, based on the range of the sampled genetic correlations, the true genetic correlation between endurance and susceptibility is likely far from unity (as only 1% of the sampled genetic correlations were above 0.8).

In the current study, tank-test-day effects were defined as random, implying the assumption that test-day effects are randomly distributed around the overall mean in each test. If the hazard rate (for the susceptible individuals) changes substantially over time during each test, these effects should ideally be fitted as fixed, to better account for potentially large temporal shifts in the hazard. However, preliminary analyses showed that fitting tank-test-day effects as fixed resulted in extreme-category problems in the cure model (results not shown). This is due to the fact that susceptibility statuses are unknown and thus inferred through the Gibbs sampler. Hence, at some point, all survivors in a specific test may be viewed as being non-susceptible (i.e., all remaining susceptible individuals die during the last test-day), resulting in extreme-category problems for the endurance trait. These problems were solved by fitting the tank-test-day effects as random [24]. Likewise, fitting the batch-tank effect for susceptibility as fixed may cause similar problems (i.e., as all individuals in a given tank at some point may be viewed as susceptible). These effects were therefore also fitted as random for the susceptibility trait in the CURE model.

The NAÏVE model is a sub-model of the CURE model when assuming 100% susceptible animals. Hence, if the NAÏVE model is the true model underlying the data, the estimated susceptible fraction is expected to approach 100%, as has been observed in simulated data sets [19]. However, in the data analyzed here, the susceptible fraction was very accurately estimated at 38% (± 1) and was never even close to the value assumed by the NAÏVE model. As previously mentioned, all challenge-tests were continued until mortality stopped for 24 h. Despite this, mortality never approached 100% in any of the challenge-tests. Thus, the study gives clear evidence for the existence of a substantial fraction of Colombian Pacific white shrimp being non-susceptible to TS.

In aquaculture breeding programs, challenge tests for infectious diseases have frequently been terminated at intermediate and often still increasing mortality. The reason for this (apart from obvious practical and economical limitations in follow-up time) is that genetic evaluations have frequently been based on survival measured as a binary trait, for which intermediate frequencies are advantageous in genetic evaluation. However, if the aim is to reduce susceptibility, rather than prolonging time until death (increase endurance), this is not optimal. Actually, terminating the test at a still increasing mortality implies that selection is shifted towards improved endurance rather than reduced susceptibility. If possible, challenge tests should therefore continue until mortality naturally ceases, as this maximizes the potential importance of susceptibility status on the recorded end-survival (and survival/censoring time). This testing strategy is thus optimal for the CURE model and will also minimize differences in ranking of selection candidates among different statistical models (SIMPLE, NAÏVE and CURE) and, thus, increase robustness of the genetic evaluations.

For classical survival models in general, a high degree of censoring is always viewed as unfavorable, as this is considered as loss of information. For cure survival models, a high degree of censoring may, however, be an advantage, provided that this to a large extent is explained by presence of non-susceptible individuals. The proposed cure model can be extended to involve single gene effects and/or genomic breeding values. For example, a cure survival model has been used to discriminate between single gene effects on incidence and latency of scrapie in sheep [25]. In Atlantic salmon, a major QTL has been identified that gives virtually complete protection against the viral disease infectious pancreatic necrosis [26], indicating that a cure survival model may be appropriate for this trait. Furthermore, the cure model can be used to account for incomplete exposure to infection in field data, i.e., the "cured" animals may be unexposed (in this case susceptibility has no heritability). If not accounted for, presence of unexposed animals could give downwardly biased estimates of the underlying genetic variance of disease resistance [27].

## Competing interests

The authors declare that they have no competing interests.

## Authors' contributions

JØ did the statistical analysis and wrote the manuscript, PM and JØ developed the statistical software to handle these models, TG and CP were responsible for recording of data and challenge-test protocols, MHY was responsible for data management and editing, MR coordinated the project and BG and THEM participated in writing the draft manuscript. All authors read and approved the final manuscript.
